# The peripheral olfactory code in *Drosophila* larvae contains temporal information and is robust over multiple timescales

**DOI:** 10.1098/rspb.2016.0665

**Published:** 2016-05-25

**Authors:** Micheline Grillet, Dario Campagner, Rasmus Petersen, Catherine McCrohan, Matthew Cobb

**Affiliations:** Faculty of Life Sciences, University of Manchester, Manchester M13 9PT, UK

**Keywords:** olfaction, temporal coding, electrophysiology

## Abstract

We studied the electrophysiological activity of two classes of *Drosophila melanogaster* larval olfactory sensory neurons (OSNs), Or24a and Or74a, in response to 1 s stimulation with butanol, octanol, 2-heptanone, and propyl acetate. Each odour/OSN combination produced unique responses in terms of spike count and temporal profile. We used a classifier algorithm to explore the information content of OSN activity, and showed that as well as spike count, the activity of these OSNs included temporal information that enabled the classifier to accurately identify odours. The responses of OSNs during continuous odour exposure (5 and 20 min) showed that both types of neuron continued to respond, with no complete adaptation, and with no change to their ability to encode temporal information. Finally, we exposed larvae to octanol for 3 days and found only minor quantitative changes in OSN response to odours, indicating that the larval peripheral code is robust when faced with long-term exposure to odours, such as would be found in a natural context.

## 1. Introduction

Peripheral olfactory coding involves responses by olfactory sensory neurons (OSNs) as part of a combinatorial code; in general, each OSN class responds to more than one odour, and each odour can activate more than one class of OSN [1]. The electrophysiological activity of individual OSNs during odour stimulation can be broadly classified into three types: excitation, inhibition, and no change from spontaneous firing activity, with excitatory and inhibitory responses showing a large quantitative range, depending on the OSN/odour combination [2]. Spike count codes provide animals with high levels of information, enabling representation of a wide variety of aspects of the stimulus. As a result, most studies of peripheral olfactory activity (e.g. [2]) have focused on spike count to describe the responses of different OSN/odour combinations.

In many sensory systems, the temporal pattern of spikes within a time window contains information beyond that conveyed by spike count [3,4]. Most explorations of temporal coding, in olfaction and in other sensory modalities, have focused on the central processing of stimuli (e.g. [4,5]). Peripheral OSNs also appear to show temporal coding [6,7]; where olfactory stimuli have a temporal structure, OSNs can respond to these temporal features [8]. In adult *Drosophila*, different OSN/odour combinations show different response latencies, suggesting that temporal coding in the periphery may constitute an additional coding dimension alongside spike count [9,10].

Little is known about how or whether the informational content of OSN responses changes over different timescales. Most electrophysiological experiments use brief periods of odour stimulation—often around 1 s. While this corresponds to one aspect of real-life olfaction (sniffing, or moving through an odour plume), animals can also be immersed in odours for much longer periods.

This paper explores two major issues in peripheral olfactory coding: the existence and significance of temporal coding, and the consistency of peripheral responses over different timescales. We studied the electrophysiological responses of the *Drosophila melanogaster* larva, which possesses only 21 pairs of unique OSNs housed in a pair of sensilla called the dorsal organs [11]. Using the Gal4-UAS system, it is possible to create larvae with a single-functional pair of identified OSNs [11]. The remaining OSNs are non-responsive, producing unmodulated spontaneous activity, while the electrophysiological activity of single-functional OSNs appears to be no different from those of wild-type OSNs [2], enabling individual OSN responses to be examined in isolation. We studied the responses of OSNs expressing Or24a and Or74a olfactory receptors. These OSNs were chosen because they respond to different ranges of odours and because Or24a is more broadly tuned than Or74a—but with some overlap [12].

We explored how much information regarding odour identity is carried by a single OSN and then investigated whether OSN activity is maintained during stimulation over four different timescales: 1 s, 5 min, 20 min, and 3 days. By combining electrophysiological data and algorithmic approaches to OSN signal content, we revealed the existence of temporal coding and its maintenance in larval OSNs over ecologically significant timescales, suggesting that this phenomenon is of significance in real-world olfactory processing.

## 2. Material and methods

### (a) *Drosophila* stocks

Stocks were maintained on a cornmeal–agar–glucose medium at 25°C under a 12 light (L) : 12 dark (D) cycle. Larvae were reared under the same conditions on a yeast paste. Single-functional OSN lines (Or24a and Or74a) were created according to the protocols outlined in [11] using lines kindly supplied by Professor Leslie Vosshall (Rockefeller University).

### (b) Electrophysiological recordings

Three-day-old larvae were immobilized with Parafilm on a moistened matchstick. A chloride-coated silver wire reference electrode was inserted into the posterior end of the larva. Borosilicate glass capillary microelectrodes with a tip diameter of less than 1 µm were filled with *Drosophila* larval ringer solution adjusted to pH 7.1 with HCl or NaOH [13]. The tip of the microelectrode was inserted into the cuticle at the base of the dorsal organ. Electrical activity was acquired using a Neurolog system (Digitimer). The differential activity from the reference and the recording electrodes was amplified and filtered (filter unit NL125 and 126) and directed to a CED micro 1401 mk II (Cambridge Electronic Design) analogue to digital converter at a sampling rate of 10 kHz. The digital signal was recorded and analysed with CED Spike2 software (v. 7.06). Spike sorting was performed off-line using Spike2. Each recording contained the activity of only one functional OSN. The activity of the functional OSN was extracted on the basis of the amplitude and waveform of the spikes and its responsiveness to odours; where the activity of other OSNs was recorded, it showed unmodulated spontaneous activity irrespective of stimulation. A principal component analysis verified that spikes belonged to the functional OSN [2].

### (c) Odour stimulation

#### (i) Odours

Butanol, octanol, 2-heptanone, and propyl acetate were from Sigma-Aldrich and of the highest purity available and were mixed with distilled water to a final concentration of 2% in 25 ml conical flasks.

#### (ii) Odour delivery system

A continuous stream of air (2.5 ml s^−1^) was directed through a flask containing distilled water and was switched to an odorant flask for the appropriate duration before being returned to the distilled water route. The exit of the delivery system was 0.5 cm from the dorsal organ. Repeated stimuli were presented with a 30 s inter-stimulus interval. The odour delivery system used polytetrafluoroethylene tubing, and odour flasks were sealed with silicone plugs. A photo-ionization detector (PID) sensor (Alphasenses) showed that odour delivery was reliable and consistent.

#### (iii) Continuous odour exposure experiments

Continuous exposure to octanol took place for periods of 5 min, 20 min, and 3 days. For the 5 and 20 min periods, larvae were exposed to a continual odour flow and continuous recordings were made of activity from the relevant OSN. For the 3-day exposure experiment, 20 µl of octanol was loaded onto a filter paper placed inside a 0.5 µl Eppendorf tube pierced by six holes and fixed to the lid of a Petri dish, the bottom of which was covered in yeast paste. The larvae fed on the paste and were unable to come into direct contact with the odour source. In the 5 and 20 min exposure experiments, the larva was stimulated before and after odour exposure in the sequence: octanol, 2-heptanone, and propyl acetate, each odour for 1 s and repeated five times. Each recording started and finished with a control stimulus (distilled water). For 3-day exposure, the sequence octanol, 2-heptanone, and propyl acetate were repeated 10 times following exposure and preparation for recording.

### (d) Classifier algorithm

#### (i) Odour decoding

Analyses were performed using MATLAB (MathWorks). A peri-stimulus time histogram- (PSTH)- classifier [14] was constructed from odour-evoked spike data. For each 1 s stimulation trial, spike times within a time window of a given duration (maximum = 6 s), starting at stimulus onset, were discretized into 50 ms bins [9] to form a response vector. A leave-one-out cross-validation approach was used, whereby the classifier was trained using data from all trials except the *i*th one (training set) and then tested on trial *i* (testing set). A template response vector for each of the four odours was obtained by averaging the response vectors for that odour to form a PSTH. For each trial, Euclidean distances between the test response vector and each template vector were computed and the template with the smallest distance was selected. If the test response vector was equidistant to two or more templates, a random selection was made from among the equidistant templates. This procedure was repeated for *i* = 1 … 5 trials and, for each odour, we calculated the fraction of trials on which it was decoded correctly. To assess whether the decoding performance for a given odour was statistically significant, we computed the distribution of performance values for all 22 larvae under the null hypothesis that the OSN was firing randomly, by generating surrogate datasets where the relationship between odour and response was randomized. One-tailed *t*-tests for each time bin were run with the Bonferroni correction. In a variant of this analysis, based on ‘sliding windows', decoding performance was computed for responses of fixed duration (200 ms) from various start times. Decoding performance was then calculated as a function of the start time.

#### (ii) Classifier: spike count

To assess the accuracy of decoding based only on spike count information, for each trial–odour combination, the elements of the corresponding response vector were randomly shuffled. This destroyed any temporal structure of the spike train, but preserved the number of spikes inside the response time window. The decoding procedure was then repeated using these shuffled data. These analyses were repeated on a fixed time window (stimulus delivery period), comparing all possible pairs of odours.

### (e) Statistical analysis

Where parametric tests were used, data distributions were first checked for normality. Analyses were performed with GraphPad, XLSTAT 2012, or SPSS.

## 3. Results

### (a) Electrophysiological responses to odour stimuli

**Figure 1.**
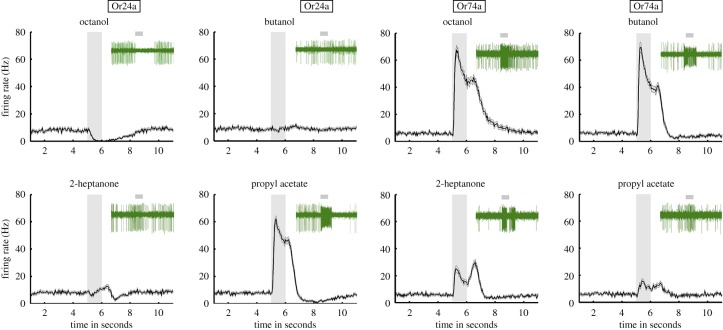
Electrophysiological responses to four odours shown by Or24a and Or74a OSNs. Graphs show means from five presentations of each odour in 22 larvae; firing activity (in hertz) is plotted in 50 ms bins. Grey bars indicate 1 s stimulus. Error bars: standard error (s.e.). Each panel includes a typical trace (green) showing the electrophysiological activity of a single OSN of that class; some of these traces show higher levels of spontaneous activity than those that appear in the pooled histograms. Mean activity in hertz (with s.d.) of OSN/odour combinations (*n* = 110 in all cases) during 1 s stimulation: Or24a/octanol = 2.85 (2.32), Or24a/butanol = 9.11 (5.88), Or24a/2-heptanone = 8.77 (6.24), Or24a/propyl acetate = 46.95 (16.84), Or74a/octanol = 50.48 (23.8), Or74a/butanol = 48.81 (24.1), Or74a/2-heptanone = 18.28 (15.4), and Or74a/propyl acetate = 11.82 (10.9). Mean number of spikes (with s.d.) of OSN/odour combinations (*n* = 110 in all cases) over 6 s of experiment (1 s stimulation and 5 s after stimulus offset): Or24a/octanol = 6.52 (6.77), Or24a/butanol = 27.90 (12.74), Or24a/2-heptanone = 23.23 (12.91), Or24a/propyl acetate = 77.26 (30.93), Or74a/octanol = 109.92 (55.77), Or74a/butanol = 85.4 (41.31), Or74a/2-heptanone = 44.00 (26.00), and Or74a/propyl acetate = 29.20 (20.40). Mean spontaneous activity rates (with s.e.) in the second before stimulation onset: Or24a/octanol = 7.5 (0.5), Or24a/butanol = 8.2 (0.5), Or24a/2-heptanone = 7.9 (0.5), Or24a/propyl acetate = 7.9 (0.5), Or74a/octanol = 5.6 (0.3), Or74a/butanol = 5.9 (0.3), Or74a/2-heptanone = 5.9 (0.3), and Or74a/propyl acetate = 5.9 (0.3). (Online version in colour.)

The *in vivo* firing activity of Or24a and Or74a OSNs (*n* = 22 for each) in response to five 1 s presentations of each of four odours—octanol, butanol, 2-heptanone, and propyl acetate—is shown in figure 1. Most OSN/odour combinations produced a unique response profile (strong excitation, weak excitation, or inhibition) that outlasted the 1 s stimulus presentation. The peak instantaneous firing rates of these larval OSNs were around 60 Hz, with most activity less than 40 Hz.

### (b) Peripheral activity contains temporal information

To explore whether the firing responses contain enough information for the individual OSNs to correctly identify each of the four odours, we used a classifier algorithm implemented in MATLAB that was trained to decode odour identity using the raw firing data. The task of the classifier algorithm was to identify which of the four odours had induced the pattern of individual OSN activity on a given trial; we termed this as a forced four-choice ‘discrimination’ task.

**Figure 2.**
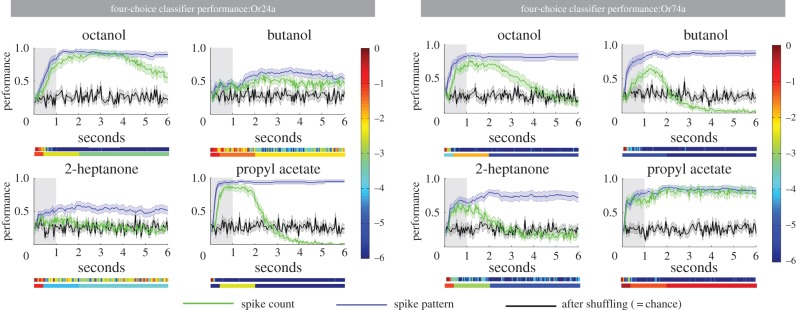
Classifier decoding performance on a four-choice discrimination task composed of responses of Or24a and Or74a, based on spike count code (green) or spike pattern code (blue). Black: performance after trial–odour combination shuffling (i.e. chance level). Shaded area around each line: s.e. Heat bars under each graph show the significance of comparisons (log_10_ *p*-values). Upper heat bar, comparisons between the spike pattern curve (blue) and performance after trial–odour combination shuffling (green); lower heat bar, comparisons for each phase of the experiment (early, middle, and late, see text).

**Figure 3.**
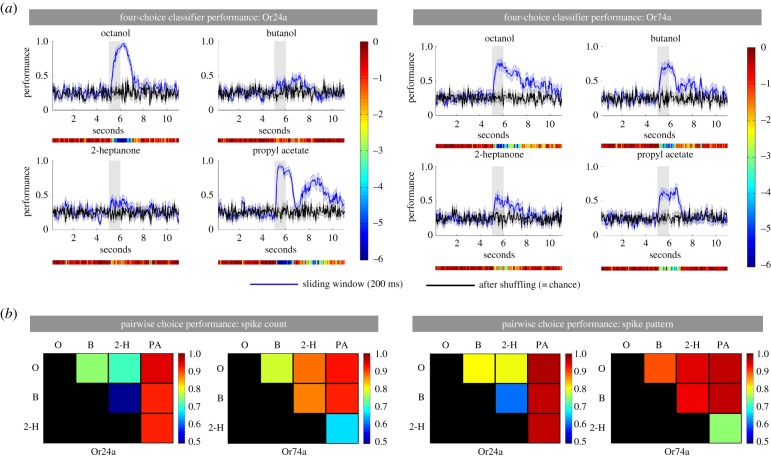
Performance of classifier using spike count and spike pattern. (*a*) Average performance over 22 larvae of the 200 ms sliding window classifier model (blue) faced with a four-choice discrimination task. Black: performance after trial–odour combination shuffling. Shaded area around curves: s.e. Heat bars under each graph show the log_10_ *p*-values of bin-by-bin comparisons between the sliding window classifier model and performance after trial–odour combination shuffling. (*b*) Decoding performance of the classifier based on spike count code (left) and spike pattern code (right) when faced with a two-choice discrimination task. The colours correspond to the performance of the model in discriminating between each pair of odours, as given by the vertical heat map.

For Or24a neurons, two of the four odours (octanol and propyl acetate) were reliably identified by the classifier with high levels of performance (perfect performance = 1.0, observed performance = 0.95 for both octanol and propyl acetate; see figure 2, blue lines). Significant above-chance levels of performance (black lines) were reached within 550 ms of stimulus onset for octanol and 150 ms for propyl acetate. Average across-larva identification performance levels for butanol and 2-heptanone were lower, but still significant (maxima = 0.66 and 0.59, respectively). For Or74a neurons, the classifier was highly effective for all four odours; significant performance was reached within 150–400 ms of stimulus onset and maximum performance of 0.80–0.87 was reached. For both OSN classes, significant performance was still obtained when the model had access only to the firing activity after stimulus offset (this was tested using a 200 ms ‘sliding window’; figure 3*a*). These classifier experiments indicate that the information contained in peripheral OSN responses is sufficient to discriminate odours with a high degree of accuracy in a two-bit classification task.

To test for the contribution of temporal information to the code, we randomly shuffled the sequence of 50 ms response time bins, thus keeping the number of spikes for each trial constant (i.e. ‘spike count’) but destroying any information in the temporal structure of the spike train (‘spike pattern’; figure 2, green lines). If there is no temporal information in the activity of the OSNs, but only spike count information, then the classifier should perform equally well with the shuffled dataset as with the original data.

After training the classifier on these time-shuffled data, we tested for significant differences in discrimination performance between the spike count code and the spike pattern code in three *ad hoc* phases of the OSN response (early = 0–400 ms; middle = 450 ms–2 s; late = 2.05–6 s—see figure 2, bottom panels). In the absence of information from the temporal pattern, spike count alone yielded significantly lower discrimination performance (*p* < 0.01) in at least one phase for all OSN/odour combinations, with the exception of Or74a/propyl acetate (figure 2 and electronic supplementary material, S1). Pairwise comparisons of the ability of the classifier to reliably identify odours reinforce this point. For example, where the number of spikes was similar (e.g. Or74a/butanol and Or74a/octanol—see legend to figure 1), the classifier showed a greater ability to reliably identify the odour when temporal information was included than when spike count alone was taken into account (figures 2 and 3*b*).

We conclude that although spike count alone can provide sufficient information for a single OSN to distinguish some odours, the responses contain additional temporal information—spike pattern—that permits substantially more reliable and more rapid odour identification.

### (c) Peripheral coding over longer timescales

**Figure 4.**
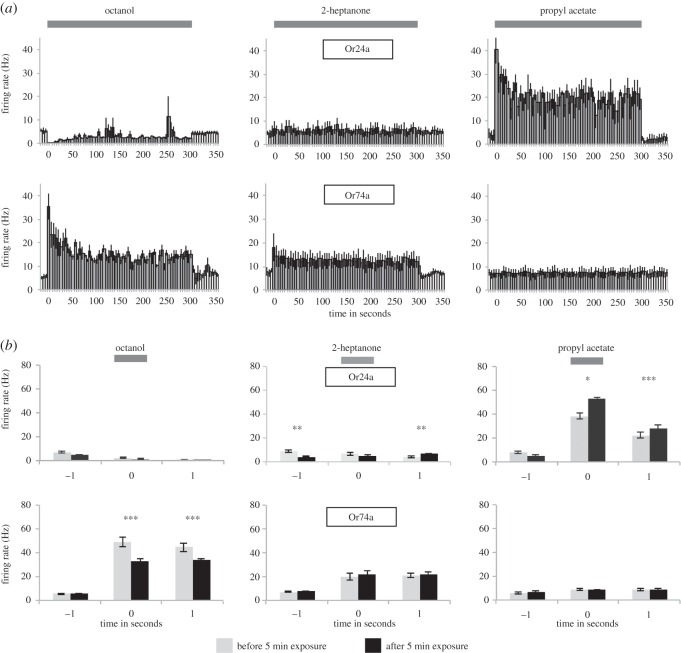
Electrophysiological responses during and after 5 min stimulation with octanol, 2-heptanone, and propyl acetate. (*a*) Firing activity of Or24a and Or74a OSNs in 5 s bins (grey; *n* = 5–6 for each OSN). Also shown are one bin before and 10 bins after stimulation (white). (*b*) Activity (spike count) of Or24a and Or74a OSNs before (grey columns) and after (black columns) exposure to octanol for 5 min. Activity shown: 1 s stimulation (‘0’, horizontal bar), 1 s before (‘−1’) and after (‘+1’) stimulation. Significant differences between before- and after-exposure values for −1, 0, and +1 are given by asterisks (*n* = 5–6 for each group).

Larvae spend virtually their whole lives in food, with their OSNs continuously stimulated by food odours. To investigate how OSNs adjust to longer periods of exposure to an odour, single-functional Or24a and Or74a larvae were exposed for 5 min to octanol, 2-heptanone, or propyl acetate and the electrophysiological activity of the functional OSN during this period was recorded (figure 4*a*). We focused on these three odours, because they represented three different chemical functional groups, and induced a range of responses in the two classes of OSN. For the sake of clarity, the 1 s period during which OSN responses were tested was termed ‘stimulation’, while longer durations were termed ‘exposure’.

Four of the six OSN/odour combinations showed a significant change in the mean firing rate over the full 5 min-exposure period compared with spontaneous activity (*p* < 0.0001, Kruskal–Wallis test with Dunn's *post hoc* comparison). The two non-significant exceptions were Or24a/2-heptanone and Or74a/propyl acetate (figure 4*a*), both of which also showed low levels of response to a 1 s stimulus (figure 1).

Or24a was strongly inhibited during the first minute of exposure to octanol (0 spikes s^−1^); it then recovered to 2 spikes s^−1^ but still showed a significant reduction in activity over the 5 min of stimulation (*p* < 0.0001, Kruskal–Wallis test). The remaining three OSN/odour pairs (Or24a/propyl acetate, Or74a/octanol, and Or74a/2-heptanone) showed significant increases in the firing rate for the full 5 min exposure, with two OSN/odour pairs showing significant declines in the level of activity following the first 60 s (Or24a/propyl acetate: 41 spikes s^−1^ at the beginning of odour exposure and only 20 spikes s^−1^ after 60 s, *p* = 0.019, Mann–Whitney; Or74a/octanol: 35 spikes s^−1^ at the beginning and 14 spikes s^−1^ after 60 s, *p* = 0.0001, Mann–Whitney). We conclude that these neurons respond continuously during long periods of odour exposure (minutes) and do not show complete adaptation.

We next explored whether 5 min exposure to a given odour altered OSN responses to subsequent 1 s stimulation with that same odour (figure 4*b*). Or24a neurons showed a significant increase in their response to 1 s propyl acetate following 5 min exposure to propyl acetate (mean firing rate = 38.3 ± 2.4 spikes s^−1^ before exposure; 52.8 ± 1.1 spikes s^−1^ after exposure, Mann–Whitney *U*-test, *p* ≤ 0.001). The spontaneous activity of Or24a OSNs was affected by 5 min exposure to 2-heptanone and propyl acetate, as shown by significant reductions in pre-stimulus (‘−1’) firing rates following exposure. A small but significant increase in the low level of firing after stimulus offset was seen after exposure to 2-heptanone. For Or74a, the only significant change in activity after 5 min exposure to an odour was seen for octanol, where exposure significantly reduced firing rates during 1 s stimulation (Mann–Whitney *U*-test, *p* = 0.004).

**Figure 5.**
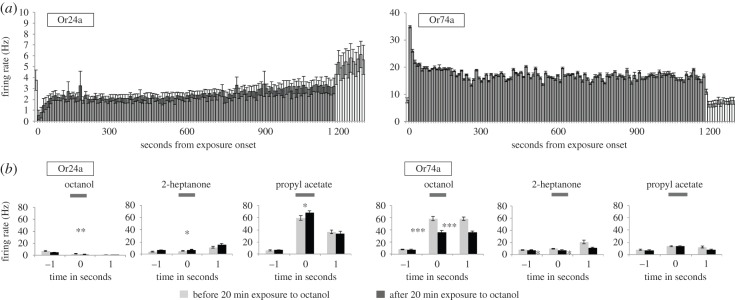
Electrophysiological responses during and after 20 min stimulation with octanol. (*a*) Electrophysiological activity of Or24a and Or74a OSNs during 20 min stimulation with octanol, in 10 s bins (grey; *n* = 5 for Or24a, *n* = 6 for Or74a). Also shown are one bin before and 12 bins after stimulation (white). Note different *y* scales. (*b*) Activity of Or24a and Or74a OSNs in response to octanol, 2-heptanone, and propyl acetate, before (grey columns) and after (black columns) exposure to octanol for 20 min. Activity shown: 1 s stimulation (‘0’, horizontal bar), 1 s before (‘−1’), and after (‘+1’) stimulation. Significant differences between before- and after-exposure values for −1, 0, and +1 are given by asterisks (*n* = 5 for Or24a, *n* = 6 for Or74a).

We next explored the specific effects of octanol on OSN activity by exposing both Or24a and Or74a OSNs to octanol for 20 min while recording their electrophysiological activity. Both OSN classes maintained the qualitative response they showed during 1 s stimulation–inhibition (Or24a) or significant excitation (Or74a; *p* = 0.002, Kruskal–Wallis test followed by Dunn's *post hoc* test; figure 5*a*; note different scales on the *y*-axis). In the seconds after the end of octanol exposure, both classes of neuron returned to pre-exposure spontaneous firing rates.

To test for any changes in the responses of the OSNs following the 20 min period of exposure to octanol, we stimulated larvae with each of the three odours in separate 1 s stimulation periods (figure 5*b*) both before and after octanol exposure. The significant reduction in the Or74a response to octanol that was observed following 5 min exposure was also seen after 20 min exposure; indeed, the effect continued into the second after the stimulus offset. Both OSN classes showed significant cross-adaption following 20 min exposure to octanol: Or24a showed small but significant increases in firing during 1 s stimulation with 2-heptanone and propyl acetate, while Or74a showed significant reductions in the firing rate in response to 2-heptanone, during 1 s stimulation and in the second after the stimulus offset.

We used the PSTH classifier to explore the performance of Or74a neurons in a forced three-choice ‘discrimination’ task (octanol, 2-heptanone, propyl acetate) following 5 and 20 min pre-exposure to octanol. Two-tailed *t*-tests showed that the discrimination performance of Or74a neurons was not significantly different before and after exposure to octanol for either 5 or 20 min (electronic supplementary material, S2). We conclude that any change in response to 1 s odour stimulation in this class of OSN following octanol exposure does not affect its ability to discriminate the three odours tested, and in particular it did not affect the ability of these OSNs to encode temporal information relating to stimulus identity.

**Figure 6.**
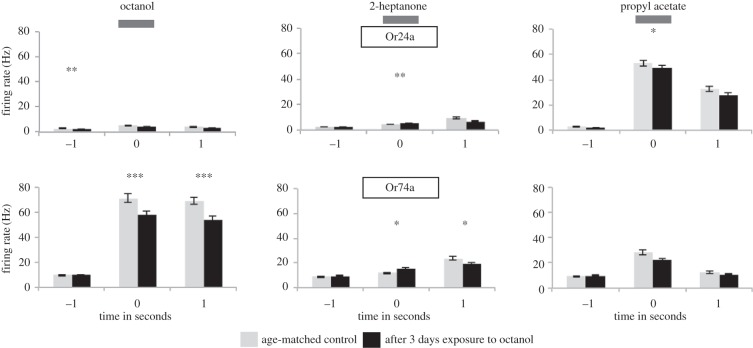
Electrophysiological responses to odours after 3 days exposure to octanol. Activity of Or24a and Or74a OSNs in response to octanol, 2-heptanone, and propyl acetate, following exposure to octanol (black columns). Age-matched larvae (grey columns) were not exposed to octanol. Activity shown: 1 s stimulation (‘0’, horizontal bar), 1 s before (‘−1’), and after (‘+1’) stimulation. Significant differences between control and experimental groups are given by asterisks (*n* (control) = 10 and *n* (octanol) = 9 for Or24a; *n* (control) = 8 and *n* (octanol) = 11 for Or74a).

Finally, we extended odour exposure time to the upper limit possible in this short-lived stage of the fly's life cycle. We reared Or24a and Or74a larvae in the presence of an octanol odour source for 3 days, and then measured their electrophysiological responses to a 1 s stimulus of octanol, 2-heptanone, or propyl acetate when compared with age-matched single-functional OSN larvae that had not been exposed to octanol (figure 6). The results were similar to those seen after 5 and 20 min octanol exposure. There was a significant reduction in the strong response to octanol in Or74a OSNs compared with control, non-exposed, age-matched OSNs, which was seen both in the 1 s stimulation and in the second following stimulus offset (Mann–Whitney *U*-test, *p* = 0.009 and <0.001, respectively). However, even after 3 days of exposure, these Or74a OSNs were still showing a firing rate of about 60 Hz during stimulation. Some cross-adaptation was seen, in the shape of a significant decline in the activity of Or74a OSNs in response to 1 s stimulation with propyl acetate (Mann–Whitney *U*-test, *p* = 0.007) and a significant decline in the response to 2-heptanone in the second after stimulus offset (Mann–Whitney *U*-test, *p* = 0.043). Although there were significant reductions in the pre-stimulus spontaneous activity of the Or24a OSN, this was only seen with octanol; in all cases, these neurons showed very low spontaneous activity (‘−1’ column). In no case was anything approaching full response adaptation seen, indicating that even on this ecologically relevant timescale, the peripheral olfactory code remains intact despite long-term odour exposure.

## 4. Discussion

Our results show that the peripheral olfactory code in *Drosophila* larvae contains both spike count and temporal information that can be used to identify odours, and that the temporal component increases the speed and accuracy of odour discrimination. Furthermore, we show that the code is robust in response to continuous stimulation with an odour—even over a period of days.

It appears that in *Drosophila* larvae the peripheral olfactory code is based on a combinatorial code consisting of the activity of single OSNs. In the adult fly antenna, OSNs are found in groups of two or three cells, housed in hair-like sensilla; if one OSN shows a sustained response, non-synaptic inhibition can occur in the other neuron [15]. We have previously found no evidence for either synaptic or ephaptic interactions between larval OSNs [2].

In agreement with previous studies (e.g. [2,12]), we found that OSN response dynamics is odour- and OR-dependent. Although it is possible that the activity of these genetically manipulated OSNs differ from that of their wild-type equivalents, there is no evidence for this [2]. Firing responses were maintained even during long exposure periods (5 and 20 min)—the overall response of an OSN to a given odour did not vary as a function of stimulus duration. Electrophysiological studies of *Drosophila* olfaction have used 0.5 or 1 s odour presentations (e.g. [2,12]). Our findings suggest that in larvae such stimuli yield electrophysiological responses that do not differ qualitatively from those seen over longer, more ecologically relevant timescales. Many OSN responses showed a second peak at or shortly after stimulus offset (figure 1); this may represent an ‘off’ response. The fact that it was seen in both OSNs and for all four odours (though not in all OSN/odour combinations) suggests this may be a fundamental feature of odour coding in these neurons.

Our classifier analysis of the electrophysiological activity of identified OSNs indicates that the peripheral olfactory code contains not only spike count information but also temporal information—spike pattern—that represents specific aspects of the olfactory stimulus. This reinforces the growing conviction that in a range of organisms peripheral olfactory codes include temporal information [6]. The time taken for the classifier to reach an odour identification performance that was close to 100% was at most a few 100 ms; after this time, discrimination capacity remained high. The sliding window analysis suggested that OSN activity contains an odour-specific signature for the entire stimulus duration, and even for some time after stimulus offset. Odour discrimination based on response temporal structure is therefore robust over time. If the larval nervous system fails to identify an odour immediately after stimulus onset, it can still exploit the sustained response to make its choice again, reducing the chance of making mistakes.

Temporal coding requires that (i) neurons convey stimulus information via spike patterns beyond that available from spike count and (ii) differences in spike pattern can modulate an animal's decisions even in the absence of a difference in spike count [16]. We suggest that temporal information in the peripheral olfactory code may be exploited by *Drosophila* larvae to make decisions about behavioural outputs. This has yet to be demonstrated in any animal.

Although there were some significant changes in the firing rates of OSNs following prolonged exposure to an odour, in no case did we observe complete adaptation—indeed, in the case of Or24a, 5 min exposure to propyl acetate significantly increased the response to that odour (figure 4*b*). In general, the responses of these two classes of OSNs were robust, in that they retained much of the spike count and spike pattern responses that characterized the responses of control OSNs. This finding may have its roots in the ecology of this species; *Drosophila* eggs are deposited on a food source (vegetable matter that is beginning to decay) by females, and under laboratory conditions larvae remain on that food source, continually bathed in food odours. In adult flies, continuous early exposure to odours causes significant plasticity in both peripheral and central structures, perhaps indicating a role for experience in the peripheral code in this species [17]. Such effects may occur in larvae, although testing this hypothesis would be challenging because rearing larvae requires continuous exposure to food and its accompanying odours.

Significant examples of cross-adaptation were seen for both OSN classes following exposure to octanol for 20 min (figure 5*b*). The two stimuli involved—octanol and 2-heptanone—must share some characteristic that is not present in propyl acetate (for which no cross-adaptation was found), and which is responsible for such effects. Further interpretation is difficult because the way that odours bind to the receptor molecule is not known, nor do we fully understand the biochemistry of olfactory receptor function.

The ability of larvae to largely maintain their OSN responses despite long-term exposure suggests that there must be substantial enzymatic activity in and around the larval OSN membrane. Putative odorant degrading enzymes have been found in adult *Drosophila* [18]; the role of such enzymes has not yet been studied in larvae.

We conclude that, at both experimental and ecologically relevant timescales, the peripheral olfactory code in *Drosophila* larvae contains temporal information. The code is robust in that the structure of odour-specific OSN responses is largely retained irrespective of experience. The next challenge will be to demonstrate the behavioural significance of temporal information in the peripheral code.

## Supplementary Material

Supplementary electronic information

## Data accessibility

All data are available at http://dx.doi.org/10.5061/dryad.2rb57.
