# Supplementary material for: The peripheral olfactory code in Drosophila larvae contains temporal information and is robust over multiple timescales
Source: Proc Biol Sci. 2016 May 25;283(1831):20160665. doi: 10.1098/rspb.2016.0665 (PMC4892805; doi:10.1098/rspb.2016.0665)
Supplement: Supplementary electronic information [file rspb20160665supp1.pdf]

	Early	Middle	Late	Early	Middle	Late
Octanol	0.2*	0.12*	0.44*	0.06	0.12*	0.11*
Butanol	0.26*	0.29*	0.79*	0.04	0.06	0.12*
2-heptanone	0.05	0.17*	0.53*	0.04	0.18*	0.28*
Propyl acetate	0.01	0.06	0.03	0.22*	0.11*	0.82*

Supplementary electronic information S1.

Mean performance distances between classifier-based curves and spike number curves shown in figure 2, based on responses of Or74a and Or24a OSNs to octanol, butanol, 2-heptanone and propyl acetate. Early = 0 – 400 ms after stimulus onset; Middle = 450 ms – 2 s; Late = 2.05 s – 6 s. * = $p < .01$, with Bonferroni's correction.

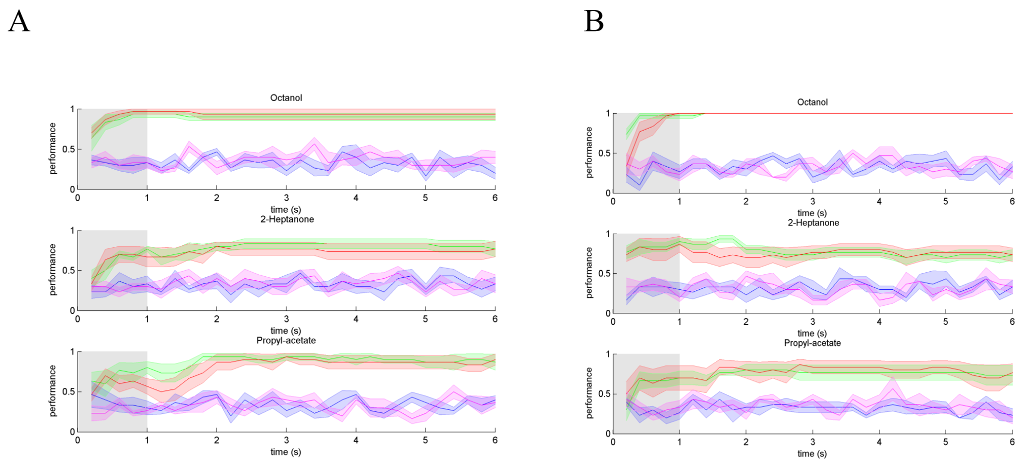

Supplementary electronic information – S2

Performance of classifier on responses after 5 and 20 minutes exposure to octanol. Decoding performance of PSTH-based classifier of Or74a OSN in discriminating three odours before (green) and after (red) continuous exposure to octanol for 5 minutes (A) and 20 minutes (B). Blue and magenta curves represent the performances of PSTH-based classifier after trial-odour combination shuffling of before and after exposure trials respectively. Shaded area around each line: SE.
